# Neuroinvasive Infection from O117:K52:H-*Escherichia coli* following Acute Pyelonephritis

**DOI:** 10.1155/2017/8976754

**Published:** 2017-11-26

**Authors:** H. Cromlin, H. Rodriguez-Villalobos, A. Deplano, T. Duprez, P. Hantson

**Affiliations:** ^1^Department of Intensive Care, Université Catholique de Louvain, Cliniques Universitaires Saint-Luc, Brussels, Belgium; ^2^Laboratory of Microbiology, Université Catholique de Louvain, Cliniques Universitaires Saint-Luc, Brussels, Belgium; ^3^Laboratory of Microbiology, Cliniques Universitaires de Bruxelles, Brussels, Belgium; ^4^Department of Neuroradiology, Université Catholique de Louvain, Cliniques Universitaires Saint-Luc, Brussels, Belgium

## Abstract

Spontaneous or nosocomial *Escherichia coli* meningitis remains rare in healthy adults but is still carrying a high mortality rate despite adapted antimicrobial treatment for susceptible strains. A 39-year-old woman was admitted to the hospital with severe subarachnoid haemorrhage complicated by acute hydrocephalus. On hospital day 10, she developed *Streptococcus anginosus* septicaemia and urinary tract infection due to a multisensitive strain of *E. coli*. This infection was successfully controlled by antimicrobial therapy. As a late complication in the neurosurgical ward (day 39), she developed fever, alteration of consciousness, and shock, leading to the diagnosis of bacterial meningitis. The culture of blood, cerebrospinal fluid, and urine grew positive for a multisensitive *E. coli*. The strain was identified as O117:K52:H, a serotype that was until now never associated with acute meningitis or brain abscesses. The source appeared to be the urinary tract with the demonstration of acute pyelonephritis. The patient died on day 94 from delayed complications of multiple brain abscesses.

## 1. Introduction

Spontaneous or nosocomial *Escherichia coli* meningitis is rarely reported in adults and usually carries a high mortality, even with adapted antimicrobial treatment [1–3]. We herein present a case of nosocomial meningitis complicated by multiple brain abscesses in a neurosurgical patient, which resulted from the haematogenous spread of an O117:K52:H-*E. coli* urinary tract infection.

## 2. Case Report

**Figure 1:**
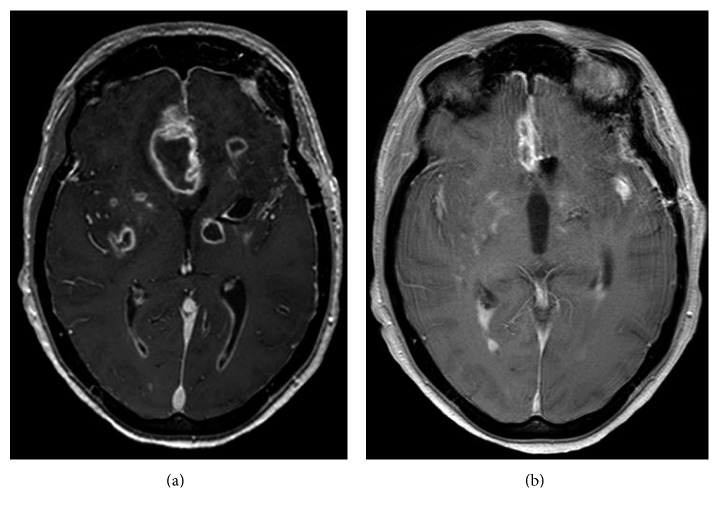
Brain MRI. Contrast-enhanced axial transverse T1-weighted view showing multiple necrotic and cystic lesions appearing as low signal intensity areas surrounded by an intensely enhanced rim corresponding to the pyogenic membrane (a). There was a regression of the lesions three weeks later (b).

A 39-year-old woman was admitted comatose, with a Glasgow Coma Scale (GCS) score of 8/15, to the intensive care unit (ICU), and brain computed tomography (CT) revealed a Fisher grade 4 subarachnoid haemorrhage (SAH) with acute hydrocephalus. Cerebral angiography demonstrated an aneurysm arising from the anterior communicating artery. She underwent surgical clipping of the aneurysm and external ventricular drainage. Due to rising intracranial pressure caused by oedema formation around a right frontal hematoma, the patient was maintained under sedation and mechanical ventilation for two weeks. Extubation was possible on day 16. Her neurological condition had finally improved to a GCS score of 13/15. Fever was noted from hospital day 4 but with negative bacteriological investigations. A multisusceptible *Escherichia coli* was first identified from the bladder catheter on day 8, but without pyuria. *Staphylococcus epidermidis* was found at CSF culture obtained from ventricular drainage, but with low cellularity (WBC count < 10 leukocytes/*µ*L). No specific antimicrobial therapy was initiated. On day 10, blood cultures grew positive for *Streptococcus anginosus*, and intravenous antimicrobial therapy was started with cefuroxime (2000 mg/day). Urinalysis revealed a moderate pyuria (294/*µ*L) and pure culture of >100,000 colony-forming units/mL of *E. coli*. Fever gradually resolved over the following days. The blood C-reactive protein (CRP) level at the beginning of antimicrobial therapy was 63 mg/L (normal value < 5 mg/L). Cefuroxime therapy was stopped after 12 days. Urine culture was sterile. Intraventricular drainage was removed on day 24, and the patient left the ICU on the same day. She was readmitted to the ICU on day 39 after sudden onset of fever (38.7°C), diaphoresis, tachypnea, tachycardia, hypotension, and altered consciousness (GCS score of 9/15). Immediate intubation was required, and norepinephrine infusion was started. Blood cultures were positive for a multisensitive *E. coli* strain, and the patient received ceftriaxone (4 g daily). Lumbar puncture revealed a purulent CSF (glucose < 3 mg/dL, lactate 29.7 mmol/L, proteins 1812 mg/dL, and leukocytes 29,105 cells/*µ*L (89% neutrophils)). Both CSF and urine cultures were also positive for *E. coli*. Pyuria was moderate (WBC count of 16 cells/*µ*L), but the blood CRP level was rising to 417 mg/L. The abdomen CT with contrast enhancement revealed wedge-shaped areas of hypoperfusion extending from the papilla to the upper part of the right and left renal cortex, together with a moderate fat infiltration around a renal cyst. The diagnosis of acute pyelonephritis appeared likely. On brain CT, ventricles were not enlarged and there was no evidence of brain abscess. By contrast, brain magnetic resonance imaging (MRI) performed five days later revealed multiple brain abscesses predominating in the grey nuclei (Figure 1). Transthoracic echocardiography was not relevant. Despite prolonged antimicrobial therapy, the patient's neurological condition only minimally improved. Blood cultures were sterile within 24 hours after initiation of antimicrobial therapy. The lumbar puncture was repeated on days 43 and 53, with negative CSF culture. Other investigations on CSF on day 53 showed leukocytes 766 cells/*µ*L, glucose 67 mg/dL, and proteins 244 mg/dL. On day 62, a surgical drainage of a large frontal interhemispheric collection (that was already present on admission brain CT and likely corresponded to a hematoma in the territory of SAH) was performed, but the culture remained sterile after seven days of incubation in aerobic and anaerobic conditions. The patient died on day 94 from delayed complications.

### 2.1. Microbiology

**Figure 2:**
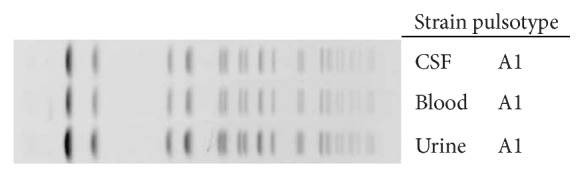
Dendogram generated by BioNumerics showing the results of cluster analysis on the basis of XbaI PFGE of *E. coli* isolates from blood, urine, and CSF.

Isolates were identified by MALDI-TOF mass spectrometer (Microflex, Bruker, Germany). Antibiotic susceptibility testing of the isolate from blood, CSF, and urine was performed by microdilution (NMIC-408 panel with BD Phoenix system, USA). According to the EUCAST clinical break points for interpretation of minimum inhibitory concentration (MIC) values, all isolates showed the same antibiotype with susceptibility to ampicillin (≤2 mg/L), aztreonam, ceftazidime, ceftriaxone and cefepime (MICs ≤ 1 mg/L), temocillin (8 mg/L), meropenem (≤0.25 mg/L), imipenem (≤1 mg/L), gentamicin (≤1 mg/L), amikacin (≤4 mg/L), cotrimoxazole (≤1/19 mg/L), ciprofloxacin (≤0.25 mg/L), piperacillin-tazobactam (≤4/4 mg/L), and amoxicillin-clavulanate (≤2/2 mg/L). Serotyping was performed in Statens Serum Institut (Copenhagen, Denmark) showing a O117:K52:H-serotype, positive for β-glucuronidase, and absence of hemolysin, eae, and verotoxins 1, 2, and 2f. To determine the genetic relatedness between the three isolates of *E. coli* (blood, CSF, and urine), isolates were analyzed via pulsed-field gel electrophoresis (PFGE) with XbaI, showing the same pulsotype (Figure 2).

## 3. Discussion

*Escherichia coli* (in particular, strains possessing the K1 capsular polysaccharide) is the most common Gram-negative organism that causes meningitis during the neonatal period. However, meningitis caused by this organism is rare in adults [1–5].

Literature data suggest that community-acquired meningitis in adults due to *E. coli* usually develops secondary to metastatic infection in patients with bacteraemia of intestinal origin [6, 7]. In a recent retrospective series of spontaneous Gram-negative meningitis, *E. coli* was the most common pathogen, and the mortality rate was still 53% [8]. Specifically, multivariate analysis identified urinary tract as focus of infection as the most important independent factor associated with a higher risk of spontaneous Gram-negative bacilli meningitis [8]. This finding is clinically relevant, since a Gram-negative bacillary etiology should be suspected in adults with spontaneous acute meningitis who have a history or a simultaneous diagnosis of bacteraemic urinary tract infection. Our patient had an asymptomatic bacteriuria with an *E. coli* isolate of community origin. The only risk factor for disseminated infection was the presence of a bladder catheter at the time the patient was hospitalized in the ICU.

Most of nosocomial meningitis cases are related to neurosurgical procedures, head trauma, or intracranial haemorrhage [5, 9].

In a retrospective analysis of 291 patients admitted with subarachnoid haemorrhage, the proportion of patients who developed meningitis/ventriculitis was 3% [10]. *E. coli* as a causative microorganism was found in 14% of cultures. The same rate of meningitis/ventriculitis was found among 202 patients with spontaneous intracerebral haemorrhage [11]. Our patient suffered from subarachnoid haemorrhage with subsequent hydrocephalus and transient need for external ventricular drainage. However, the catheter had been removed 14 days before the development of meningitis.

The symptoms of *E. coli* meningitis are nonspecific, but sustained episodes of fever and a marked deterioration of consciousness are usually observed [2]. While third-generation cephalosporins remain the empiric treatment of choice for Gram-negative meningitis, the emergence of extended-spectrum beta-lactamases- (ESBL-) producing *E. coli* isolates is becoming a therapeutic challenge [7, 12]. The benefit of intraventricular/intrathecal antimicrobial therapy is not clearly demonstrated.

To the best of our knowledge, this is the first isolation of this serotype associated with meningitis. *E. coli* O117 strains are associated with septicaemia and diarrhea [13–15]. Verocytotoxin-producing *E. coli* O117:H7 was identified in UK as a causative agent of sexually acquired infections in men who have sex with men (MSM) [16]. The majority of these infections were produced by verocytotoxin-producing isolates. However, in the present case, a particular nonmotile O117:K52:H-*E. coli* nontoxigenic isolate was detected. In this way, some authors suggest that the O-antigen form may be important for the pathogenicity of O117 isolates [17]. At present, no more cases of infection related with this particular serotype have been reported.

In the present observation, septicaemia originated from a urinary tract infection that relapsed despite a previous course of adapted antibiotic treatment.
